# Bending actin filaments: twists of fate

**DOI:** 10.12703/r/12-7

**Published:** 2023-03-21

**Authors:** Mitsutoshi Nakamura, Justin Hui, Susan M Parkhurst

**Affiliations:** 1 Basic Sciences Division, Fred Hutchinson Cancer Center, Seattle, WA, USA 98109

**Keywords:** Cytoskeleton, actin, actin bending, actin helicity, chirality, Septins, Anillin, α-Actinin, Fascin, Cofilin, unconventional myosins, IQGAP, L-R asymmetry

## Abstract

In many cellular contexts, intracellular actomyosin networks must generate directional forces to carry out cellular tasks such as migration and endocytosis, which play important roles during normal developmental processes. A number of different actin binding proteins have been identified that form linear or branched actin, and that regulate these filaments through activities such as bundling, crosslinking, and depolymerization to create a wide variety of functional actin assemblies. The helical nature of actin filaments allows them to better accommodate tensile stresses by untwisting, as well as to bend to great curvatures without breaking. Interestingly, this latter property, the bending of actin filaments, is emerging as an exciting new feature for determining dynamic actin configurations and functions. Indeed, recent studies using *in vitro* assays have found that proteins including IQGAP, Cofilin, Septins, Anillin, α-Actinin, Fascin, and Myosins—alone or in combination—can influence the bending or curvature of actin filaments. This bending increases the number and types of dynamic assemblies that can be generated, as well as the spectrum of their functions. Intriguingly, in some cases, actin bending creates directionality within a cell, resulting in a chiral cell shape. This actin-dependent cell chirality is highly conserved in vertebrates and invertebrates and is essential for cell migration and breaking L-R symmetry of tissues/organs. Here, we review how different types of actin binding protein can bend actin filaments, induce curved filament geometries, and how they impact on cellular functions.

## Introduction

Directional forces within cells are necessary for a wide spectrum of developmental events including cytokinesis, left/right (L-R) asymmetry, *Xenopus* convergent extension, Zebrafish epiboly, *C. elegans* ventral enclosure, *Drosophila* germband extension/retraction and dorsal closure, and mouse palate formation^1–15^. The generation of such intracellular forces depends heavily on the dynamic organization and regulation of actomyosin networks and other cytoskeletal proteins. Actin and myosin form a number of different functional assemblies within cells, including supracellular actomyosin cables, cortical-junctional actomyosin, apical-medial actomyosin, and basal actomyosin networks^14,16–21^. Actin filaments and myosin form distinct dynamic structural geometries within these different assemblies based of their spatial organization and connectivity, which leads to different proficiencies in force generation^1,3–5,7,10–16,21^.

**Figure 1.**
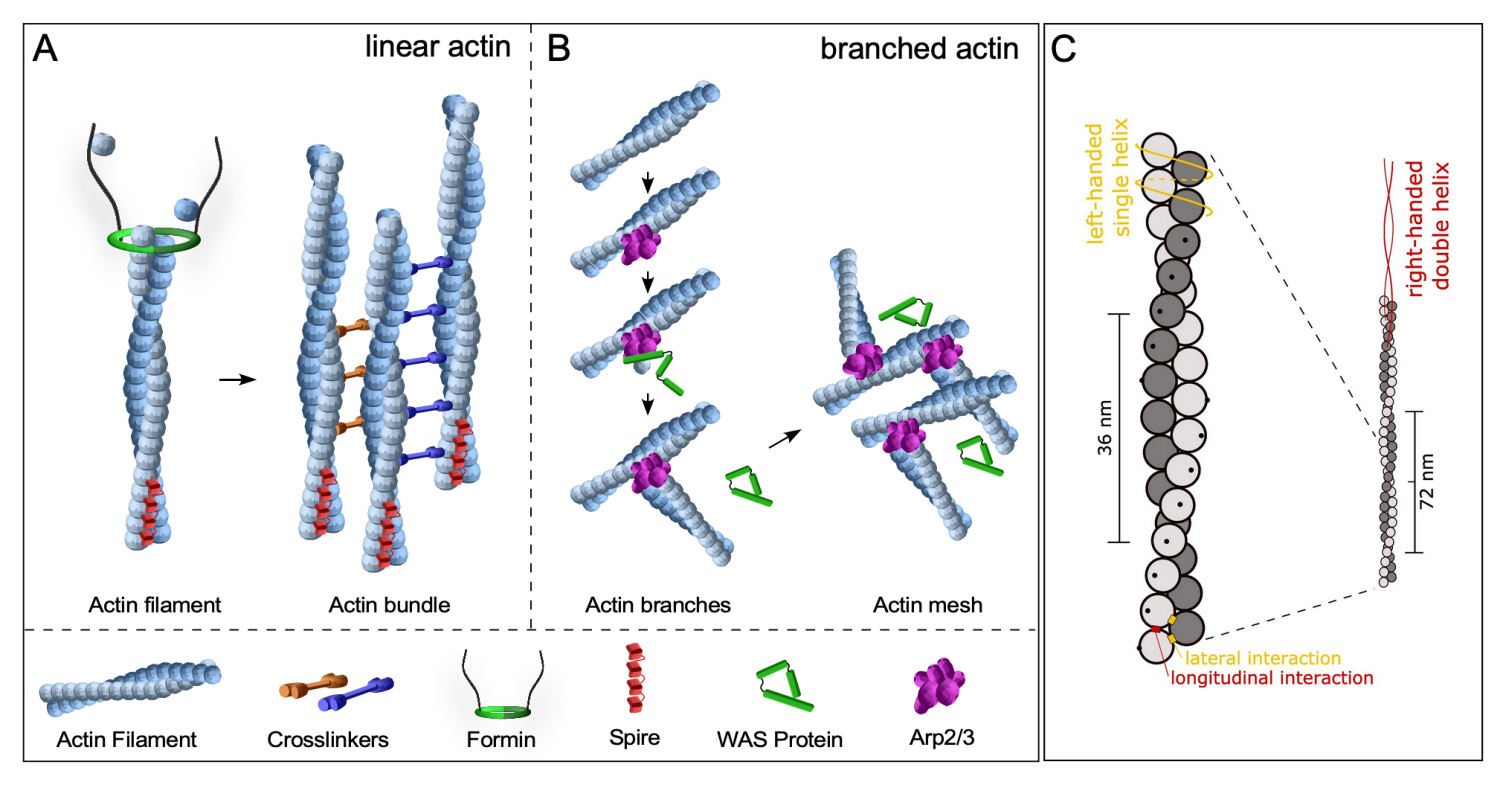
Actin filament formation and helicity. (**A**) Schematic diagram showing *de novo* linear actin filament formation through nucleation promoting factors such as Formins and Spire. Formins act via actin dimer stabilization and processive movement with the elongating filament (fast-growing) barbed end, whereas Spire forms a prenucleation complex containing up to four actin monomers. Actin crosslinkers bind to the sides of actin filaments to aid in the formation of filament bundles. Adapted from Hui *et al.* (2022), Cells **11**(18): 2777; https://doi.org/10.3390/cells11182777^39^. (**B**) Schematic diagram showing *de novo* branched actin filament formation via the Arp2/3 complex and Wiskott-Aldrich Syndrome (WAS) family proteins. Arp2/3 binds to the sides of preexisting linear actin filaments and nucleates branched actin networks upon activation by WAS family proteins. Adapted from Hui *et al.* (2022), Cells **11**(18): 2777; https://doi.org/10.3390/cells11182777^39^. (**C**) Actin filaments are helical. Schematic diagram of an actin filament that is generated from actin monomers (spheres with black dots showing orientation of monomers). The double helix actin filament has a pitch of 72nm. The filament can be seen as either a left-handed, one-start, single helix (orange line) or a right-handed, two-start, double helix (red line). Reprinted from Jegou *et al*. (2020), Semin. Cell Dev. Biol. 102:65-72. doi: 10.1016/j.semcdb.2019.10.018, with permission from Elsevier^34^.

Actin is often characterized as being assembled into linear filaments through the action of *de novo* linear actin nucleation factors such as formins and Spire^22–25^ (Figure 1A). These linear actin filaments can be organized into different assemblies through bundling and/or cross-linking proteins, allowing them to perform functions including filopodia formation. Linear actin filaments also serve as the scaffold for the formation of branched actin through the action of *de novo* branched actin nucleation factors including Wiskott-Aldrich Syndrome family proteins and the Arp2/3 complex^22,26–33^ (Figure 1B). Branched actin meshes are associated with cell events including lamellipodia formation and cell migration.

Actin filaments themselves are also inherently helical and this helicity has been demonstrated to provide the filament with its mechanical strength and play an important role in facilitating cell chirality and L-R asymmetric morphogenesis^34^ (Figure 1C). Interestingly, this intrinsic twisting is maintained during formin mDia1-dependent actin filament elongation as a result of mDia1 rotating along the filament axis as it actively incorporates actin molecules^35^. The canonical actin filament double helix has a pitch of 72nm and completes 13.9 turns per micrometer. Notably, computational modeling predicts that filaments under 200pN of tension can untwist approximately 2° to elongate the filament^36^. In a separate study, tension on actin filaments was shown to enhance the binding affinity of myosin II motors^37^. Additionally, it was observed that Arp2/3-mediated branching occurred more frequently on the convex side of bent filaments^38^. Multiple studies have now shown cooperative and preferential binding to different actin binding proteins (ABPs) depending on the physical conformation of the actin filament^34,40–42^. Intriguingly, a recent study showed that the nucleotide state of actin filaments can have a major impact on the rigidity of the filament and influence its twisting and bending^42^. The specific nucleotide state in the presence of bending forces can additionally influence the spatial rearrangements of actin molecules and change the helical twist of actin filaments as predicted by computer simulations^43^. Therefore, there exists a complex feedback loop between the physical stresses applied to an actin filament, its conformation, and the combination of ABPs bound. This complex system of forces and ABPs can dictate the conformation of a given actin filament and/or the organization/geometry of actin filament assemblies to properly sense and react to cellular and environmental events.

In addition to their roles in assembling linear and branched actin filament assemblies, a number of ABPs have recently been shown to alter the properties of actin filaments, triggering them into curved geometries. Here, we review the ability of different ABPs—alone or in conjunction with other ABPs—to alter the conformation of actin filaments and/or the geometry of actin filament assemblies, and their effect on cellular and developmental events.

### IQGAP/calponin homology proteins

**Figure 2.**
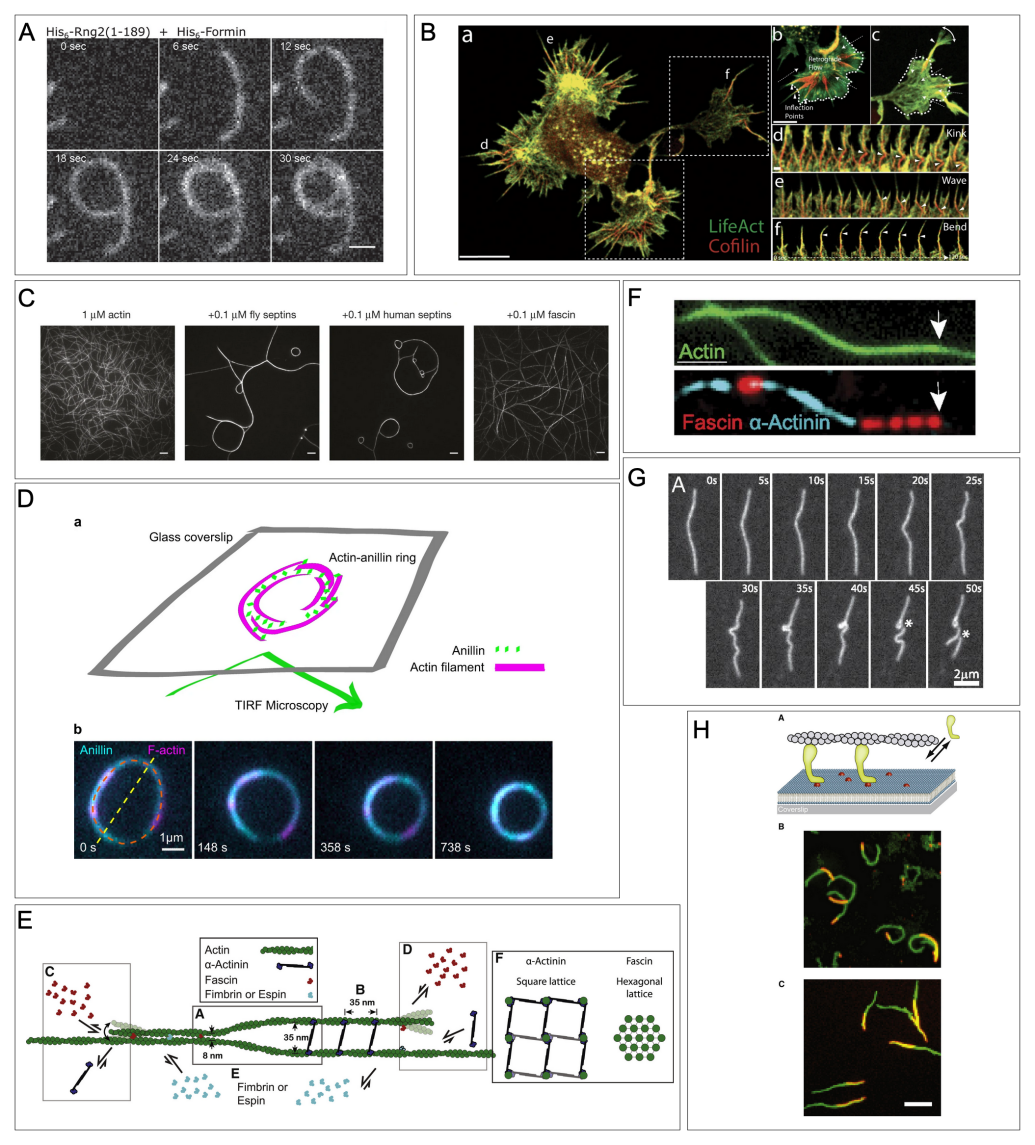
Actin bending by different types of actin binding proteins. (**A**) Actin filaments polymerized by formin bend in the presence of the curly region (1-189 aa) of IQGAP. Scale bar: 1 μm. Reprinted from Palani *et al*. (2021), eLife 10:e61078. doi: 10.7554/eLife.61078^48^. (**B**) Single slice image of Primary E18 rat hippocampal neurons labeled with actin (green) and Cofilin (red)(a). Maximum intensity projections of lower and upper dashed square areas in a, respectively (b-c). Time-lapse images showing different types of actin conformation (Kink, Wave, and Bend) in a (d-f). Scale bars: 5 μm (a and b), 500 nm (d). Reprinted from Hylton *et al.* (2022). Nat Commun. 13(1):2439. doi: 10.1038/s41467-022-30116-x^59^. (**C**) *in vitro* polymerized F-actin co-incubated with no protein, *Drosophila* Septins, human Septins, or Fascin. Both *Drosophila* and human Septins bundle and bend F-actin. Scale bars: 5 μm. Reprinted from Mavrakis *et al.* (2014). Nat. Cell Biol. 16(4):322–34. doi: 10.1038/ncb2921, with permission from Springer Nature^72^. (**D**) Schematic diagram showing TIRF microscopy for F-actin and Anillin imaging (a). Time-lapse images showing actin ring contraction by Anillin (b). Reprinted from Kučera *et al.* (2021). Nat. Commun. 12(1):4595. doi: 10.1038/s41467-021-24474-1^73^. (**E**-**F**) Schematic diagram showing spaces and shapes of bundled actin by Fascin and α-Actinin (**E**) and *in vitro* polymerized F-actin co-incubate with Fascin and α-Actinin showing that Fascin and α-Actinin localization is segregated (**F**). Reprinted from Winkelman *et al.* (2016) Curr. Biol. 26(20):2697–2706. doi: 10.1016/j.cub.2016.07.080, with permission from Elsevier^74^. (**G**) Time-lapse images for F-actin buckling. Reprinted from Murrell *et al*. (2012), Proc. Natl. Acad. Sci. USA 109(51): 20820–20825. doi: 10.1073.pnas, with permission from PNAS^75^. (**H**) Schematic diagram showing F-actin and membrane-tethered myosins in the myosin gliding assays (a). Maximum intensity projections from 10 min (b) and 20 min (c) time-lapse movies. Green and orange actin filaments show later and initial time points, respectively. Myo1C induces anti-clockwise actin filament swirling, but Myo1A does not. Scale bar: 5 μm. Reprinted from Pyrpassopoulos *et al.* (2012) Curr. Biol. 22(18):1688–92. doi: 10.1016/j.cub.2012.06.069, with permission from Elsevier^76^.

The IQGAP (IQ motif-containing GTPase-activating proteins) protein family are scaffolding proteins with multifarious interactions that allow them to facilitate actin dynamics at the cell membrane. They have been implicated in dynamic actin processes such as cell migration^44–46^. IQGAPs possess recognizable protein motifs, including calponin homology domains (CH), coiled-coil domains (CC), and GTPase R domains (GRD), that contribute to their modulation of actin assemblies^44,45^. The CC domain has been demonstrated to bind to Ezrin, a cytoskeleton-plasma membrane anchor, and the GRD domain transiently protects the Cdc42 small GTPase in its activated state by reducing GTP hydrolysis. These two domains allow the IQGAP family to localize to the plasma membrane and sustain the interaction between the WASP branched actin nucleator and the Arp2/3 complex through its CH domain, thereby promoting branched actin nucleation^47^. In addition to binding WASP, the CH domain has been shown to bind actin filaments with high affinity and, strikingly, to bend actin filaments to a high curvature^48^. In a recent study, Palani and colleagues creatively used the CH domain of Rng2 (fission yeast IQGAP ortholog) to show that it was capable of bending actin filaments to form ring structures^48^ (Figure 2A). In a series of *in vitro* assays, the authors demonstrated that membrane-tethered His-tagged CH domain not only bent actin filaments, but consistently bent filaments anti-clockwise. This observation suggests a level of inherent chirality in actin filament bending because of bound CH domain-containing proteins. Interestingly, in a separate study, Harris and colleagues showed that the conformation of an actin filament can have a stark effect on the binding kinetics of a tandem CH domain reporter^49^. The authors measured the dwell time of tandem CH domain reporters on actin filaments in the presence of actin stabilizing drugs (phalloidin and Jasplakinolide) and other actin binding proteins (Drebrin, Heavy meromyosin, Cofilin) to show that the conformation of the actin filament can alter how well the reporter bound to actin filaments. As actin filaments are inherently helical, some bound proteins can change the pitch of actin and induce twisting or bending forces to give rise to high-order actin assemblies such as a ring. The bending or twisting of actin filaments can sterically influence the localization of other ABPs or result in preferential binding of specific ABPs (e.g., the Arp2/3 complex)^38^. Thus, there is a striking reciprocity in which the binding of ABPs alters the conformation of actin filaments, while the conformation of actin filaments affects which ABPs can bind.

### Cofilin

Cofilin is a member of the ADF/cofilin family of actin binding proteins that are known for severing and/or depolymerizing actin filaments, thereby regulating actin filaments through the generation of dynamic actin disassembly^50–53^. Notably, Cofilin has been shown to sever bent actin filament regions more efficiently^54^. When Cofilin binds to actin filaments, the helical half-pitch of the decorated actin filaments (referred to as “cofilactin” filaments) changes: 37 nm for bare actin filaments and ~27 nm for cofilactin filaments^55–57^. The shorter helical pitch and the transition from bare to cofilactin filaments led to accelerated actin severing. However, saturated cofilactin filaments are no longer severed cofilin-dependently and are more flexible^50,52,58^. While examining actin bundles within filopodia at neuronal growth cones using cryo-electron tomography, Hylton and colleagues recently showed that cofilactin filaments associate with curvature of the actin bundles, thereby regulating filopodia structure and dynamics^59^. Interestingly, this cofilactin filament conformational change results in an exclusion of the Fascin actin-crosslinking protein from cofilactin bundles, leading to less organized actin filament bundling, the bending of filopodial protrusions, and a disruption of filopodial structure and functions (Figure 2B)^59^. These results led the authors to propose that cofilin-mediated bending of actin filaments in filopodia would provide enhanced filopodia environmental searching/sensing to aid in the efficiency of targeted neural outgrowth^59^.

### Septins

Septins are a family of highly-conserved GTP-binding proteins that form hetero-oligomeric complexes that polymerize into filaments and play important functions in many cellular processes, including cytokinesis, cell polarity, epithelial wound healing, and membrane remodeling, and when mis-regulated, are associated with neurodegenerative diseases and cancer^60–67^. These dynamic Septin structures function at the cell cortex where they associate with membrane composed of specific curvatures or lipid compositions, as well as with actin and microtubule cytoskeletal networks composed of specific architectures or properties^60,61,64,65,68–71^. Given their various oligomeric assemblies and prime cortical localization, Septin complexes have been implicated in both membrane- and cytoskeleton-associated functions: i.e., aiding in the spatial segregation of cell compartments, crosslinking/bending cytoskeletal filaments, serving as scaffolds, and/or serving as membrane-bound diffusion barriers. Septins are divided into four different classes that assemble into hetero-hexamer or -octamer complexes that form higher-order structures, including rings, gauzes, and collars^64,69,77^. Oligomeric Septin filaments have been demonstrated to cooperate with actin filaments in dynamic structures such as the cytokinetic ring^78^. Septins often colocalize with bundled and curved actin filaments: *in vitro* assays have shown that both fly and human Septin hexamers can bundle actin filaments and bend them into various curved geometries such as rings and loops with diameters ranging 3-18μm^72^. Intriguingly, both fly and human Septins decorated actin filament bundles and rings without forming full rings themselves *in vitro^72^*, suggesting that Septins are not acting as a template for actin filaments to form a ring, but are instead bending actin filaments as they bind to the side of the filaments. Septin-mediated actin filament bending results in greater curvatures of the filaments (Figure 2C). Using the rapid cellularization of the developing *Drosophila* embryo, Mavrakis and colleagues went on to demonstrate the function of Septin-induced curved actin filaments *in vivo*. They found that actin filaments formed bar-like structures in *septin* mutant embryos, rather than the ring-like structures observed surrounding the nucleus in wildtype embryos. Thus, both *in vitro* and *in vivo* assays conducted in this study strongly suggested that Septins play a role in bending and circularizing actin filaments by binding to the side of the filaments to facilitate formation of higher-order structures. Intriguingly, using cryo-EM, Mendonça and colleagues recently described a human Septin hetero-hexamer filament that tends to bend at its center where two Septins form a homodimeric interface^79^. This opens the possibility that when this bent Septin hetero-hexamer oligomerizes and binds to actin, it bends actin to form a ring structure.

### Anillin

Anillin is a crosslinker of both Septins and actin that contains a pleckstrin homology domain allowing it to anchor to cell membranes^80^. As a prominent Septin binding protein, Anillin has been shown to also play a critical role in cytokinesis, particularly in organizing and anchoring the cytokinetic ring at the plasma membrane^78,81^. Although there is yet to be direct evidence showing how Anillin bends or curves actin filaments, recent studies have demonstrated that Anillin can generate contractile force^73^ and that Anillin is required to properly orient Septin filaments at the cytokinetic ring^78^. In a nicely executed series of experiments, Kučera and colleagues showed that Anillin actively moves linear actin filaments and facilitates actin ring constriction by promoting force generation in a myosin-independent manner (Figure 2D)^73^. Similar to Septins, Anillin formed actin rings *in vitro*, demonstrating its potential to bend actin filaments. In a second study conducted by Arbizzani and colleagues, the fission yeast Anillin ortholog, Mid2, was shown to participate in aligning Septin filaments parallel to the cytokinetic ring^78^. Using polarized fluorescence microscopy, they found that Septins remained randomly oriented when Anillin was deleted. Given the role of Septins in bending actin filaments (discussed above), Anillin may do more than simply bundling actin: Anillin may act to generate and facilitate organizational changes in Septins and actin to modulate the tunability and optimization achieved by actin assemblies.

### α-Actinin and Fascin

α-Actinin and Fascin are actin crosslinkers that can influence the mechanical characteristics of actin filaments/bundles and how they respond to different applied stresses or geometrical constraints^74,82,83^. Actin crosslinkers can dynamically regulate the stiffness of actin filament bundles and the stability of actin networks through bundling or bridging actin filaments in many cellular processes, including filopodia formation and focal adhesion assembly^84–86^. While both α-Actinin and Fascin can bundle individual actin filaments to form tightly packed parallel filaments, the structures of these resulting actin filament bundles are different. A combination of TEM and *in vitro* actin bundling assays showed that actin filaments bundled by α-actinin have ~35 nm space between each filament and form square lattice structures, whereas actin filaments bundled by Fascin have ~8 nm space and form a hexagonal lattice structure^74,83^ (Figure 2E). *In vitro* actin bundling assays also showed that α-Actinin and Fascin exhibit mutually exclusive localization on actin filaments^74,83^ (Figure 2F). Because these actin crosslinkers generate different spacings among actin filaments and can be present on different regions of these assemblies, actin filaments must bend to accommodate these differences^74,83^. Consistent with *in vitro* assays, localization of α-Actinin and Fascin is also mutually exclusive within cells. For example, Fascin localizes in the distal portion of filopodia, whereas α-Actinin localizes in the proximal region^87,88^. While the biological function of local actin bending by α-Actinin and Fascin has not been directly addressed, their coordination is thought to be important for cell stiffness^89^.

### Myosins: conventional and unconventional

Myosins are a large family of motor proteins that move on actin filaments and are required for many cellular processes including muscle contraction, intracellular transport, endocytotic pathways, and left-right (L-R) asymmetric morphogenesis^90–97^. Among 15 different classes of myosins, Myosin II is known as conventional myosin and has been well-studied with respect to its ability to generate force through the concerted movement of actin filaments. Myosin II is bipolar and generates contractile force by binding to and sliding parallel actin filaments past each other. Surprisingly, recent studies showed that bipolar Myosin II can also bind to a single actin filament^75,98,99^. In this case, one end of the bipolar myosin protein is oriented towards the barbed end of the actin filament, whereas the other end is oriented towards the pointed end. As myosin preferentially moves towards the barbed end of actin filaments, the head oriented towards the barbed end will move faster than the head oriented towards the pointed end. In response to these different velocities either in the same direction or in opposing directions, the region of the actin filament between the myosin filament ends will bend/buckle (Figure 2G).

Unconventional myosins are actin-based motor proteins that are grouped into distinct classes based on their motor and tail domains, which provide different structural and functional characteristics^94,100–105^. Their conserved N-terminal motor domains bind to actin and their class-specific C-terminal domains regulate the selection of and binding to cargos or other cell components^94,100–105^. Unconventional myosins have been studied with a focus on their function as cargo transporters, molecular anchors (to the actin cytoskeleton or plasma membrane), tension sensors, actin crosslinkers, signal transducers, and mediators of membrane-cytoskeleton interactions needed for a wide range of cellular processes, including endo-/exo-cytosis, organelle and membrane trafficking, cell motility, and L-R asymmetry^94,96,97,102–104,106^. Type I unconventional myosins are tethered to the membrane and regulate cellular processes such as membrane tension and intracellular trafficking in cells^107^. While examining the role of type I unconventional myosins in the generation of L-R asymmetry in different *Drosophila* tissues, Lebreton and colleagues eloquently showed that two of these, Myo1D and Myo1C, generate *de novo* directional twisting of cells and organs in opposite directions to establish chirality^108–110^. Intriguingly, the motor domain of Myo1D, but not that of Myo1C, produced counterclockwise circular motility of actin filaments *in vitro*. In contrast to *Drosophila* Myo1C, mammalian Myo1C does produce a counterclockwise circular motility of actin filaments *in vitro* (Figure 2H)^76^. A subsequent study examining L-R asymmetric morphogenesis in *Drosophila* showed that individual cells in the gut exhibit a directional chiral shape, and that such cell chirality is regulated by MyoID^111^. Intriguingly, this individual cell chirality induces the L-R asymmetric morphogenesis of the *Drosophila* gut^111^. Overall, directional actin motility regulated by type I Myosin may generate the force to deform the cell into a chiral shape that subsequently determines L-R asymmetry in the tissues.

## Conclusions

Actin networks must dynamically change their configurations to generate the forces needed within cells for many of their essential biological processes^1,3–5,7,10–16^. The ability of proteins to bend individual or groups of linear actin filaments is emerging as an important contributor to the dynamic regulation of higher-order actin assemblies required for cellular processes such as the formation of an actin ring, cell migration, and endocytosis^48,72,73,112,113^. Bending actin filaments directly regulates actin network geometries, provides directional actin branching for ABP binding (i.e., Arp2/3 prefers binding to the convex side of actin filaments^38^), provides regions for more efficient severing^54^, and can be used to generate force by unbending. In addition, a recent study suggests that nucleotide-dependent modifications of the actin filament conformational landscape influences the binding specificity of ABPs^42^. Intriguingly, α-Actinin, IQGAP, and unconventional myosins also induce directional actin filament movements such as counterclockwise swirling. This actin-dependent cell chirality is observed in a wide range of animals from *Drosophila* to mammals^57,111,114–121^. While much of the evidence for actin bending and its biological consequences to date comes from studies using *in vitro* systems, recent studies using cells and model organisms revealed that cell chirality has important roles in processes such as cell migration and L-R asymmetric organ development^97,106,122,123^. We still have much to learn about how altering the conformation of actin filaments by bending them into curved geometries affects the functions of actin assemblies, results in chirality, and the means by which it is used to achieve precise developmental outcomes. In addition, it remains unclear how ABP binding induces actin filament bending and directional movement of actin filaments. The combination of cryo-EM, super-resolution microscopy, cutting-edge live-imaging techniques, single-cell RNAseq, and computer simulations can aid in further understanding the mechanistic insights and revealing the mysteries still surrounding the role of actin bending.
